# Functional Meta-Analysis of the Proteomic Responses of Arabidopsis Seedlings to the Spaceflight Environment Reveals Multi-Dimensional Sources of Variability across Spaceflight Experiments

**DOI:** 10.3390/ijms241914425

**Published:** 2023-09-22

**Authors:** Gbolaga O. Olanrewaju, Colin P. S. Kruse, Sarah E. Wyatt

**Affiliations:** 1 Molecular and Cellular Biology Program, Ohio University, Athens, OH 45701, USA; go123019@ohio.edu; 2 Department of Environmental and Plant Biology, Ohio University, Athens, OH 45701, USA; 3 Los Alamos National Laboratory, Bioscience Division, Los Alamos, NM 87545, USA; krusec@lanl.gov

**Keywords:** spaceflight, gravitropism, Arabidopsis, proteomics, meta-analysis, TMT, BRIC hardware, International Space Station

## Abstract

The human quest for sustainable habitation of extraterrestrial environments necessitates a robust understanding of life’s adaptability to the unique conditions of spaceflight. This study provides a comprehensive proteomic dissection of the Arabidopsis plant’s responses to the spaceflight environment through a meta-analysis of proteomics data from four separate spaceflight experiments conducted on the International Space Station (ISS) in different hardware configurations. Raw proteomics LC/MS spectra were analyzed for differential expression in MaxQuant and Perseus software. The analysis of dissimilarities among the datasets reveals the multidimensional nature of plant proteomic responses to spaceflight, impacted by variables such as spaceflight hardware, seedling age, lighting conditions, and proteomic quantification techniques. By contrasting datasets that varied in light exposure, we elucidated proteins involved in photomorphogenesis and skotomorphogenesis in plant spaceflight responses. Additionally, with data from an onboard 1 *g* control experiment, we isolated proteins that specifically respond to the microgravity environment and those that respond to other spaceflight conditions. This study identified proteins and associated metabolic pathways that are consistently impacted across the datasets. Notably, these shared proteins were associated with critical metabolic functions, including carbon metabolism, glycolysis, gluconeogenesis, and amino acid biosynthesis, underscoring their potential significance in Arabidopsis’ spaceflight adaptation mechanisms and informing strategies for successful space farming.

## 1. Introduction

The spaceflight environment is highly unique and challenging for terrestrial life, being radically different from Earth due to multiple factors such as microgravity, cosmic radiation, and the absence of a conventional diurnal cycle. Moreover, the constraints imposed by spacecraft or the space station, such as limited space, isolation, and specific growth systems, further contribute to the environmental peculiarities [1,2]. These spaceflight environmental conditions dramatically influence plants’ growth, development, and physiological responses, which are essential for life support systems in space. On Earth, gravity is a constant, directional cue that affects various plant physiological processes, including gravitropism, phototropism, and mechanical resistance. However, in the microgravity environment of spaceflight, these cues are disrupted, necessitating adaptation [3]. Studies have revealed altered growth patterns, with root and shoot orientations significantly differing from those observed under Earth’s gravity [4,5]. Spaceflight also exposes plants to cosmic radiation, primarily composed of highly energetic particles. While Earth’s magnetic field and atmosphere protect terrestrial life from the harmful effects of this radiation, such protection is absent in space. Cosmic radiation can induce DNA damage, oxidative stress, and other cellular damage, necessitating robust repair and defense mechanisms in space-faring plants. Notwithstanding these challenges, plants, being highly plastic organisms, have demonstrated remarkable resilience and adaptability to the spaceflight environment [6,7,8]. Key to these adaptations are changes at the molecular level, including genomic, transcriptomic, proteomic, and metabolomic modifications.

The need to understand how plants adapt to the spaceflight environment is driven by the expanding horizons of human space exploration and the prospect of establishing permanent human colonies on other planets. As we venture farther into the cosmos, plants will play a critical role in providing food and recycling air and waste [6]. This necessity puts forth an imperative for comprehensive studies on how the spaceflight environment impacts plant biology at various levels, from the cellular to the organism. Most of our current understanding of plant responses to the spaceflight environment has been gleaned from genetic and transcriptomic studies, which have shed light on various plant adaptation mechanisms. However, these studies provide a predominantly gene-centric view and do not fully capture the complexity of biological responses to spaceflight conditions. Proteins, however, being the effectors of most life processes, offer an additional layer of complexity beyond genes and transcripts. Proteins catalyze biochemical reactions, provide structural support, participate in cellular signaling, and perform a multitude of other roles. As such, proteins represent a higher level of life organization than transcripts, more accurately reflecting the functional state of a cell or an organism. Notably, gene and transcript levels do not necessarily predict protein abundance due to multiple regulatory steps, including translation efficiency and protein degradation [9,10]. Kruse et al. [11] identified this discordance and low correlation between RNA transcripts and proteins in their spaceflight Arabidopsis experiment. Paul et al. [12] and Ferl et al. [13] also noted this discordance and an organ-specific (leaf and root) response of Arabidopsis proteome to the spaceflight environment. Therefore, examining the proteomic responses of plants in spaceflight is fundamental to gaining a comprehensive understanding of biological systems, including plants under spaceflight conditions.

Despite the clear significance of proteomics to plant space biology, investigations into the proteomic responses of plants to spaceflight are surprisingly limited, with only three studies being published so far and a fourth in the publication process [13,14]. Each of these studies has provided valuable insights into how the Arabidopsis plant responds at the proteome level to spaceflight conditions. However, each of these studies is constrained by their relatively small sample size, limiting their statistical power and leaving room for further investigation. Furthermore, these studies, conducted independently, have used different methodologies and spaceflight hardware. Such variations could lead to differences in the observed proteomic responses, further complicating the interpretation of individual studies. Here, a meta-analysis approach is particularly valuable. It allows for the synthesis of data across studies, increasing the statistical power, and providing a more robust understanding of the overall proteomic responses to spaceflight. By aggregating these existing plant proteomic data from spaceflight experiments, we aim to identify consistent patterns of proteomic responses in Arabidopsis under spaceflight environmental conditions.

While transcriptomic studies of plant responses to spaceflight have significantly advanced our understanding, proteomic analyses remain comparatively underdeveloped in this unique area of research. The logistical complexities and high costs associated with spaceflight experiments have often limited the repeatability of such studies, leading to substantial variability across available datasets. Moreover, the scarcity of publicly accessible proteomic metadata further hampers direct comparisons and comprehensive meta-analyses. In light of this, our study aims to answer a pivotal question: ‘How can we leverage the limited existing proteomic data to provide a more robust and comprehensive understanding of the proteomic adaptations of Arabidopsis to spaceflight conditions?’ By conducting a meta-analysis of the limited but invaluable proteomic datasets that are currently available, this study aims to identify the key proteins and pathways implicated in Arabidopsis’ response to spaceflight. We hypothesize that the meta-analysis of the four existing proteomic datasets will reveal consistent patterns of proteomic adaptations in Arabidopsis under spaceflight conditions, identifying key proteins and pathways that are differentially regulated in response to the space environment. This endeavor not only aims to bridge the current gap between transcriptomic and proteomic studies in spaceflight plant biology but also to set the stage for future experiments that could have broader implications for long-term human space missions.

## 2. Results

### 2.1. Curating the Proteome of Different Arabidopsis Spaceflight Experiments

**Figure 1:**
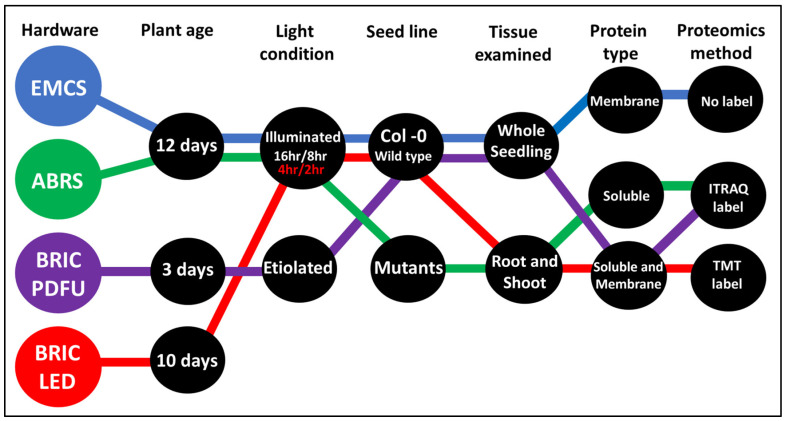
Experimental parameters for the 4 Arabidopsis spaceflight proteomics studies. Each vertical set of bubbles represents experimental parameters identified above the bubble cascade. The colored threads pass through bubbles that correspond to the dataset’s experimental parameters. Blue bubbles and thread represent the EMCS dataset; green represents the ABRS dataset; purple represents the BRIC PDFU dataset; and red represents the BRIC LED dataset.

Unlike transcriptomics studies of plants in spaceflight, which had over 15 datasets curated in the NASA GeneLab data repository [15], only four comprehensive proteomics studies have so far been conducted on plants in spaceflight aboard the International Space Station (ISS) in four different hardware. These are the European Modular Cultivation System (EMCS) [14], the Advanced Biological Research System (ABRS) [13], the Biological Research In Canister–Petri Dish Fixation Unit (BRIC PDFU) [11], and the Biological Research In Canister–Light-Emitting Diode (BRIC LED) hardware. Raw proteomics datasets (.RAW) for BRIC PDFU (also known as BRIC 20) and BRIC LED spaceflight experiments were accessed from the NASA GeneLab data depository with accession numbers GLDS 38 (https://osdr.nasa.gov/bio/repo/data/studies/OSD-38, accessed on 9 April 2023) and OSD 522 (https://osdr.nasa.gov/bio/repo/data/studies/OSD-522/preview/w5Q5ElZE-Wy6DDS1ZFzeRjBAPuKss-x0, accessed on 16 July 2023), while the ABRS dataset was accessed from the PRIDE Archive proteomics data repository with the accession number PXD001179 (https://www.ebi.ac.uk/pride/archive/projects/PXD001179, accessed on 16 July 2023). The EMCS experiment dataset was not deposited in any accessible data repository, but the ready-analyzed peptide and protein count data were attached as a Supplementary Materials to the primary publication [14]. The environmental conditions were similar for all the experiments, with exposure to Low Earth Orbit (LEO) microgravity aboard the ISS and cosmic radiation (0.2 to 0.5 mGy) [16]. The light exposure for non-etiolating experiments was around 75 Wm^−2^ of photosynthetically active radiation (PAR), with variation in the illumination cycles, plant age, seed lines, tissue examined, protein type, and peptide labeling methods (Figure 1).

The experimental parameters, unlike the environmental conditions, varied significantly among the four datasets and are responsible for significant variability between the datasets. Except for the BRIC PDFU dataset, whose seedlings were 3 days old post-germination (PG), the duration of the other spaceflight experiments (from seed to seedlings) was within a similar range (10–12 days PG). BRIC PDFU had no light exposure with the seedlings grown in the absence of light and gravitational cues, while the other datasets had varying illumination cycles—BRIC LED (4 h of light/2 h of dark), ABRS, and EMCS (16 h of light/8 h of dark). The ABRS experiment worked on three Arabidopsis green fluorescent protein (GFP) reporter gene lines, which are the alcohol dehydrogenase promoter (Adh::GFP), the synthetic auxin response element composed of five AuxRE elements (DR5r::GFP), and the CaMV35s promoter (35s::GFP) [13], while the other experiments were on the wild-type Columbia (*Col*-0) seed lines. Both ABRS and BRIC PDFU datasets examined the proteome of the whole Arabidopsis seedling while the EMCS and BRIC LED datasets examined the organ-specific proteome. The quantitative proteomics for the EMCS proteomics dataset had no isotopic label; both ABRS and BRIC PDFU utilized the Isobaric tag for relative and absolute quantitation (iTRAQ), and the BRIC LED utilized the Tandem Mass Tag (TMT).

### 2.2. Principal Component Analysis

**Figure 2:**
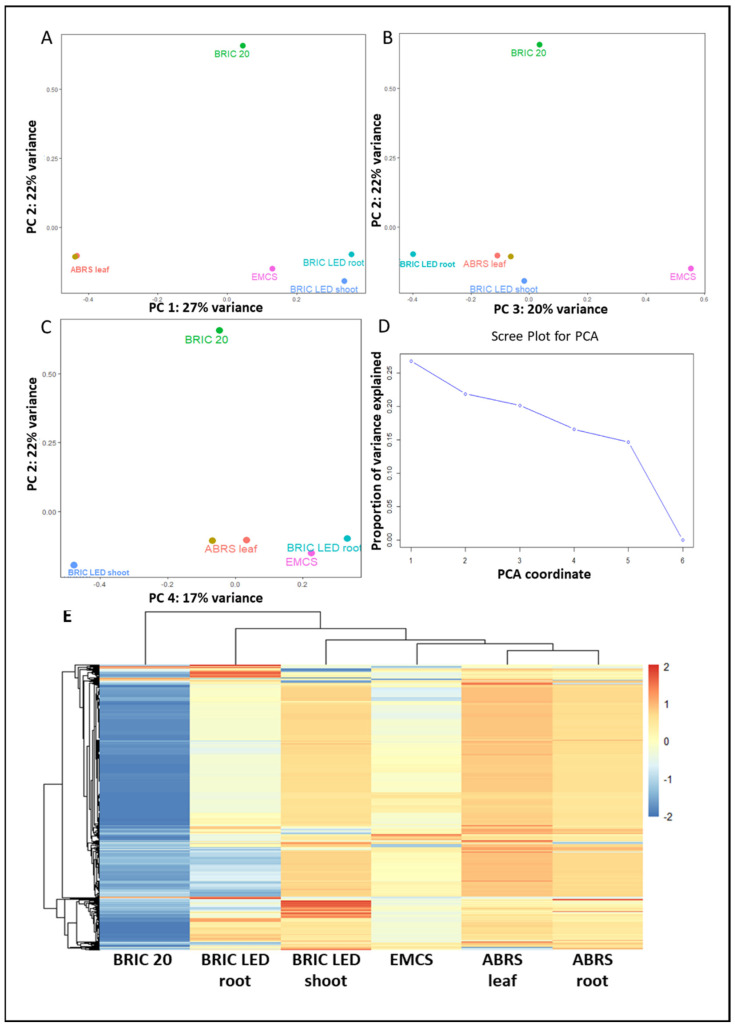
Variability and dissimilarity of the 4 proteomic datasets. (**A**) Principal component analysis depicting the percentage of explained variance by principal components 1 and 2. Each point on the graph represents each Arabidopsis spaceflight proteomics dataset. (**B**) Principal component analysis depicting the percentage of explained variance by principal components 2 and 3. (**C**) Principal component analysis depicting the percentage of explained variance by principal components 2 and 3. (**D**) Scree plot depicting the principal factor analysis of the PCA. (**E**) Euclidean hierarchical clustering measure of dissimilarity between the 4 datasets. Each point on the heat map represents individual proteins in the dataset. The color gradient represents log2 fold change (L_2_FC_protein_), with red depicting more abundant proteins in spaceflight than on Earth and blue depicting less abundant protein in spaceflight than on Earth.

To identify the degree of similarity and variations introduced by the different experimental parameters to the four proteomics datasets, we conducted a transposed principal component analysis (Figure 2A–C), which provided insights into how the individual proteins co-vary across samples. The scree analysis identified four significant (>15%) explainable variations between the datasets (Figure 2D).

The principal components 1–4 explained just 26.76%, 21.85%, 20.15%, and 16.58%, respectively, of the variability noted among the four datasets, and this indicates that the determinant of the proteome response of the Arabidopsis plant to the spaceflight environment is multi-dimensional and the data do not have a single dominant direction of variability. In other words, there is no single PC that significantly captures most of the variance in the data, but instead, the variability is spread out over several different orthogonal (independent) directions. However, the PC1 indicates that 26.76% of the variability is introduced by the different spaceflight hardware (Figure 2A). Both the ABRS leaf and root datasets are clustered together and are far away from the rest of the datasets. Similarly, the BRIC LED root and shoot datasets are also clustered near each other and distinctly from the other datasets. PC2 indicates that the seedling age and hardware lighting conditions contributed about 21.85% of the variability among the four datasets (Figure 2A), with the BRIC PDFU experiment pattern of protein expression being segregated away from the rest. The BRIC PDFU dataset was conducted in the absence of light and on 3-day-old Arabidopsis seedlings [14]. PC3 seems to indicate that the quantitative proteomics label techniques account for 20.15% of the noticed variability (Figure 2B). The EMCS dataset, which had no isotopic label, was clustered away from the rest of the dataset, which had either the iTRAQ or the TMT labels. However, PC3 also distinguished the BRIC LED root dataset from the rest, which might be an indicator of additional contributing factors to the explained variance by PC3. Likewise, the PC4, which accounted for 16.58% of the variability among the dataset, does not appear to have a definite pattern of distribution along the experimental parameters (Figure 2C). The scree plot (Figure 2D) indicates that 14.66% of the variability is accounted for by other dimensional factors. The Euclidean hierarchical clustering of the log2 fold change in each protein within each dataset indicates that the BRIC PDFU dataset was the most diverged (Figure 2E). All the differential analysis datasets are available in Supplementary File S1.

### 2.3. The Direct Intersection of the Four Proteomics Datasets

A direct intersect analysis of the four datasets enables us to conduct subgroup classifications and identify proteins that are common to particular datasets, hence hinting at the relevance of the protein to Arabidopsis response to the spaceflight environment. Intersection analysis also enables us to assess the generalizability of the results for further probing and identifying high-priority target proteins. Common to the four datasets are the GLYCINE-RICH PROTEIN and the GLYCINE DECARBOXYLASE P-PROTEIN 1 (GLDP1) located in the chloroplast and involved in mRNA binding, PATELLIN-1 (PATL), which is a member of the family of regulators of auxin-mediated PIN1 relocation and plant development [17], the PLASMA-MEMBRANE-ASSOCIATED CALCIUM-BINDING PROTEIN 1 (PCAP1) involved in damage-associated molecular patterns (DAMPs) that act as endogenous signals to activate the plant immune response [18], and a LEUCINE-RICH REPEAT RECEPTOR-LIKE KINASE 1, a key membrane-bound regulator of abscisic acid early signaling in Arabidopsis [19] (Table 1).

**Figure 3:**
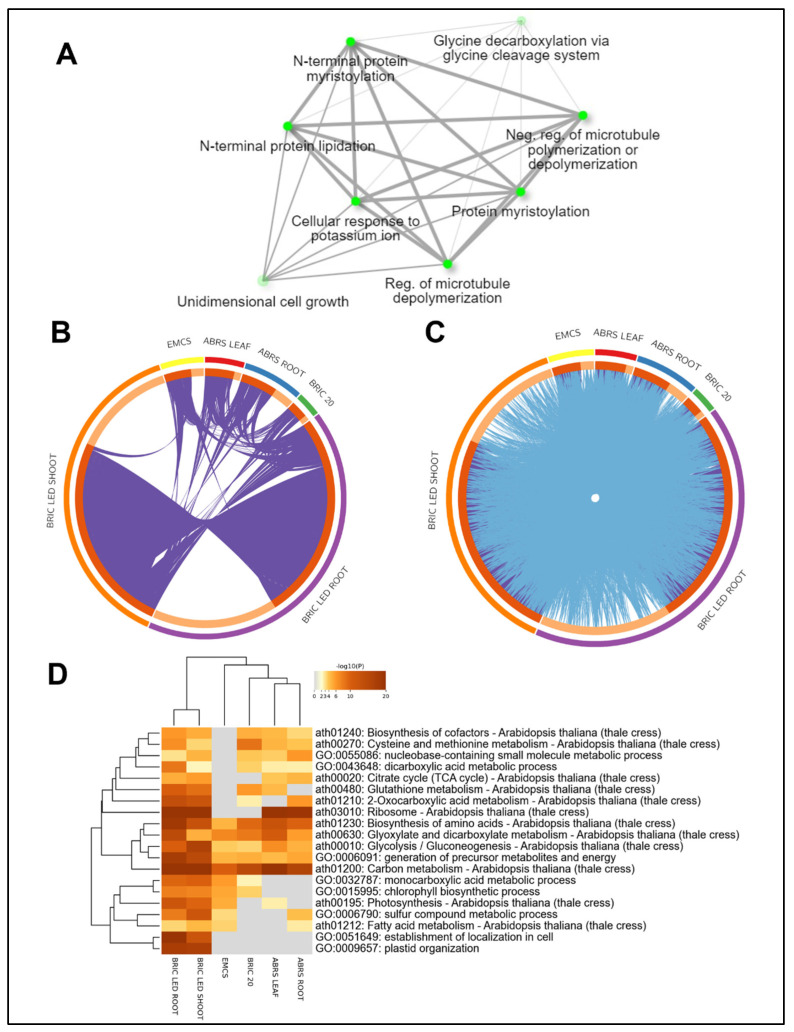
Differentially abundant proteins in spaceflight across the four datasets. (**A**) Protein–protein network interaction analysis of the five proteins common to all the datasets. Bigger nodes with deeper colors indicate pathways that are highly significant, while the thickness of the connecting lines indicates the strength of the overlap. Analysis was performed using the ShinyGO 0.76 [20]. (**B**) Overlap between the four datasets only at the individual protein expression level, where purple curves link identical proteins. (**C**) Overlap between the four datasets including the shared term level, where blue curves link proteins that belong to the same enriched ontology term. The inner circle represents protein lists, where hits are arranged along the arc. Proteins that hit multiple lists are colored in dark orange, and proteins unique to a list are shown in light orange. Analysis was performed using Metascape [21]. (**D**) Heat map of the enriched ontology term shared by the four datasets. The colored gradient indicates the significant expression of proteins involved in the GO term.

A protein–protein network analysis indicates that these proteins interact with each other. The interacting pathways are major protein modifications via lipidation and myristylation, regulation of microtubule polymerization, and unidimensional growth (Figure 3A). The intersect analysis of the four datasets also revealed that despite the limited overlap between differentially abundant proteins present in the datasets (Figure 3B), the four datasets shared substantial involvement in similar Arabidopsis metabolic pathways (Figure 3C), such as the carbon metabolism pathway, the glycolysis and gluconeogenesis pathway, the biosynthesis of amino acids, and the glyoxylate and dicarboxylate metabolism pathway (Figure 3D).

**Figure 4:**
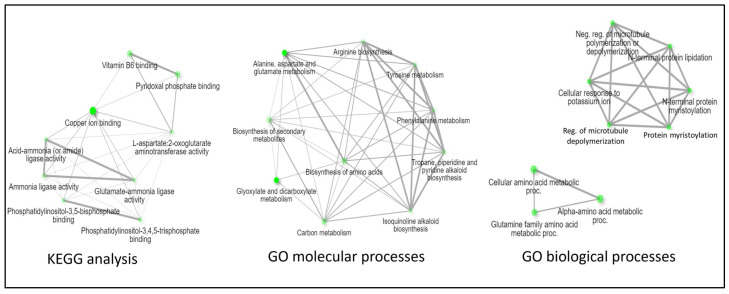
Protein–protein network interaction analysis of the 10 proteins common to the EMCS, BRIC PDFU, and BRIC LED datasets. The protein–protein interaction analysis was viewed from the KEGG analysis (**left**), molecular functionality (**middle**), and biological functionality perspectives (**right**). Nodes with deeper colors indicate pathways that are highly significant, while the thickness of the connecting lines indicates the strength of the overlap. Analysis was performed using the ShinyGO 0.76 [20].

Excluding the ABRS dataset based on the mutant Arabidopsis seed lines used in the experiment, an intersect analysis of the EMCS, BRIC PDFU, and BRIC LED datasets indicated ten common proteins among the three datasets (Table 1). Protein–protein network analysis of the shared proteins indicated that they are involved in metabolic pathways such as the biosynthesis and metabolism of several amino acids, carbon, and secondary metabolites. The molecular functionality of the 10 proteins indicates highly enriched involvement in phosphatidylinositol-3,4,5-triphosphate binding, L-aspartate: 2-oxoglutarate aminotransferase activity (a glutamate dumper), ammonia ligase activity, and copper binding (Figure 4). The GO biological processes analysis was like those observed in the intersection of all four datasets, with the proteins highly enriched in the protein myristoylation pathway and regulation of microtubule polymerization.

### 2.4. Comparing BRIC PDFU and BRIC LED Datasets: Impact of Light Conditions

**Figure 5:**
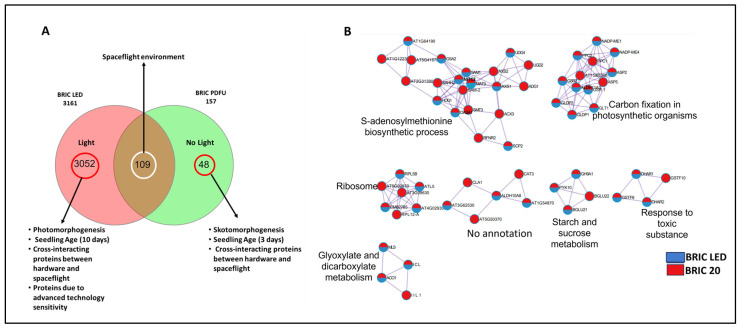
Differentially abundant proteins in the BRIC PDFU and BRIC LED datasets. (**A**) Venn diagram of the intersection of the two datasets. Arrows indicate our interpretation of the protein distributions. Venn diagram is generated by InteractiVenn (http://www.interactivenn.net/) accessed on 15 July 2023. [25]. (**B**) Protein–protein interaction network and MCODE components identified in the intersection of both datasets. The module that is labeled “No annotation” had no modular name generated by Metascape. Analysis was performed using Metascape [21].

Both the BRIC PDFU and the BRIC LED spaceflight experiments were conducted by the same research groups, and both datasets integrated transcriptomics and proteomics studies. The major difference between these two datasets is the incorporation of LED illumination into the BRIC LED hardware, which provided the Arabidopsis seedlings grown in the BRIC LED hardware with light cues. The BRIC PDFU seedlings were etiolated, being completely grown in the absence of light cues in spaceflight. Another difference is the age of the seedlings analyzed and the quantitative proteomics label used for the protein analysis (Figure 1). Despite the absence of light in the BRIC PDFU hardware, light-related genes were expressed in its transcriptomics dataset [11]. Hence, to investigate how Arabidopsis plants integrate light signaling with other spaceflight environmental conditions, we evaluated the intersections of proteins expressed in the BRIC PDFU and BRIC LED datasets and identified 109 common proteins (Figure 5A, Supplementary File S2). The BRIC LED dataset is significantly larger than the BRIC PDFU dataset, and this might be due to the incorporation of the high-field asymmetric waveform ion mobility spectrometry (FAIMS) to the LC-MS/MS analysis of BRIC LED proteomics and the use of the TMT label instead of the ITRAQ. The addition of FAIMS and the usage of TMT over the ITRAQ labeling technique have been reported to significantly increase targeted proteomics’ sensitivity [22,23,24]. Hence, we believe that while the proteins at the intersection of both datasets and those unique to the BRIC PDFU dataset are true representatives of their inferences, proteins unique to the BRIC LED may contain some artifacts (Figure 5A). Despite having proteins that are unique to the BRIC PDFU, the GO enrichment terms of these proteins are not unique to BRIC PDFU; they are common to both datasets. An application of Metascape’s protein–protein interaction enrichment analysis and Molecular Complex Detection (MCode) to the proteins at the intersection of both datasets revealed that they are enriched in pathways involved in Ribosomal activities, S-adenosylmethionine biosynthetic process, carbon fixation in photosynthetic organisms, glyoxylate and dicarboxylate metabolism, starch and sucrose metabolism, and in response to toxic substance (Figure 5B). Proteins at the intersection of the BRIC PDFU and BRIC LED datasets are predominantly expected to be responses to the spaceflight environment and devoid of hardware interference (Figure 5A).

**Figure 6:**
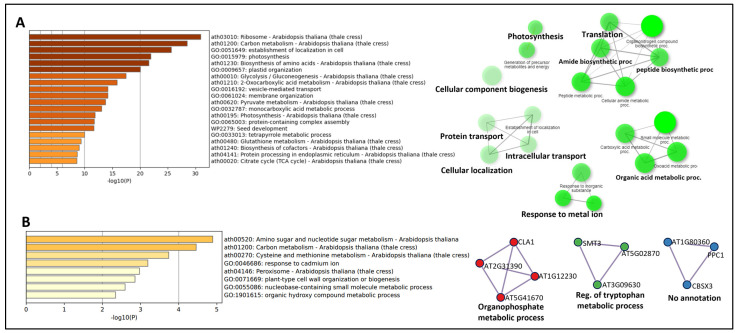
GO term enrichment of proteins unique to BRIC PDFU and BRIC LED. (**A**) Heatmap (left) and protein–protein interaction map (right) of the most significantly enriched pathways of proteins unique to BRIC LED. (**B**) Heatmap (left) and protein–protein interaction map (right) of the most significantly enriched pathways of proteins unique to BRIC PDFU. The color gradient of the heat map indicates the *p*-value of the proteins in the enriched pathway. Analysis was performed using ShinyGO v0.76 and Metascape.

The enrichment ontology of the unique proteins in the BRIC LED datasets (Figure 5A) revealed that the proteins are significantly enriched in the ribosome, carbon metabolism, establishment of cellular localization, photosynthesis, and plastid organization (Figure 6A). The protein–protein network interaction analysis also indicated that the proteins are clustered into six KEGG pathways, which are photosynthesis, cellular component biogenesis, translation, protein transport, organic acid metabolism, and response to inorganic substances (Figure 6A). Proteins unique to the BRIC LED are suggested to be involved in photomorphogenesis. Contrary to this, proteins unique to the BRIC PDFU are suggested to be involved in skotomorphogenesis, and an enrichment ontology of these proteins revealed that they are significantly enriched in pathways involved in amino sugar and nucleotide metabolism, peroxisome, and plant cell wall organization (Figure 6B). The protein–protein interaction network analysis revealed that they are clustered into 3—the organophosphate metabolic process, the regulation of tryptophan metabolic process, and an unidentified cluster (Figure 6B). See Supplementary File S2 for the list of the unique proteins.

### 2.5. Eliminating Microgravity from the Comparison

**Figure 7:**
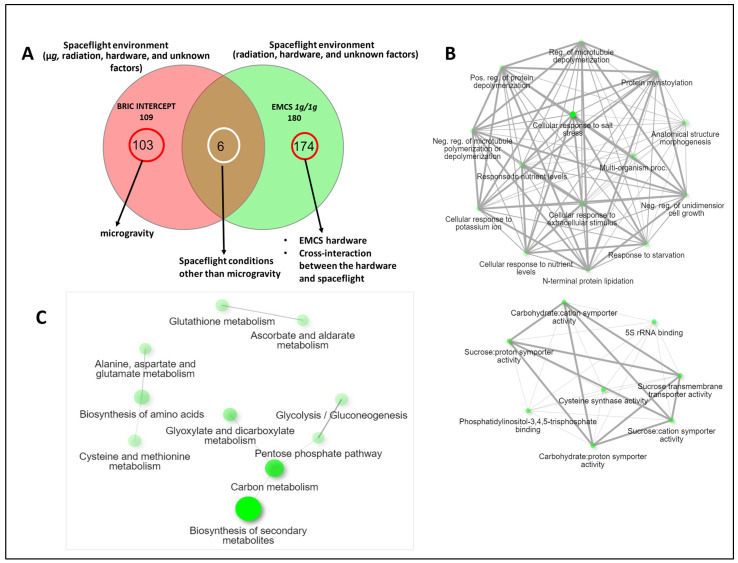
Comparison of the BRIC intercept and the ECMS 1 *g*/1 *g* datasets. (**A**) Venn diagram of the intersection of the two datasets generated by InteractiVenn [25]. Arrows indicate our interpretation of the protein distributions. (**B**) Protein–protein interaction network analysis of the biological (top) and molecular (bottom) GO term enrichments of the intersection proteins. These proteins are suggested to reflect the response to spaceflight environmental conditions other than microgravity. (**C**) Protein–protein interaction network analysis of the molecular GO term enrichments of the proteins unique to the BRIC intercept dataset. These proteins are suggested to reflect Arabidopsis response to spaceflight microgravity alone. Node sizes and colors indicate pathways significance while the thickness of the connecting lines indicates the strength of the overlap. Analysis was performed using ShinyGO 0.76 and Metascape.

Mazars et al. [15] in their EMCS experiment also generated a 1 *g* spaceflight control dataset, which eliminated microgravity from the spaceflight environment using a centrifuge running at 1 *g* aboard the ISS. We compared this dataset (referred to as EMCS 1 *g*/1 *g*) to the BRIC LED and BRIC PDFU intersection datasets in an attempt to isolate the effects of spaceflight microgravity from the comparison (Figure 7A). The six proteins found at this intersection are the SUCROSE TRANSPORT PROTEIN (SUC1), CYSTEINE SYNTHASE (OASA1), PCAP1, BGLU21, which is a scopoline-hydrolyzing beta-glucosidases, MLP 34, and the RIBOSOMAL PROTEIN L5 (ATL5) (Supplementary File S2). Protein–protein network analysis of the biological functionality of these six proteins revealed a single cluster of interrelated biological processes pathways’ highly enriched in metabolic pathways involved in cellular response to extracellular stimuli such as salt stress, potassium ion, nutrient level, and starvation (Figure 7B). An analysis of the molecular functionality revealed the enrichment of pathways such as 5S rRNA binding, carbohydrate–cation symporter activity, cysteine synthase activity, and phosphatidylinositol-3,4,5-triphosphate binding (Figure 7B).

Unique to the BRIC intercept dataset are 103 proteins, which are clustered into three and are highly enriched in pathways involved in carbon metabolism, the pentose phosphate pathway, glycolysis, glutathione, ascorbate, and aldarate metabolisms, and the biosynthesis of amino acids (Figure 7C). These proteins are listed in Supplementary File S2.

## 3. Discussion

### 3.1. The Determinant of the Arabidopsis Proteomic Response to the Spaceflight Environment Is Multidimensional

The overarching goal of this meta-analysis is to assess the degree of dissimilarity and identify the significant sources of variability between the four proteomic datasets, thereby discerning the key influences on the plant’s proteomic responses to the unique environment of space. Our analysis indicated that the experimental conditions, namely, the spaceflight hardware, seedling age (experimental duration), hardware lighting conditions, and peptide labeling methods, were substantial contributors to the observed variability, reaffirming the multifaceted nature of spaceflight effects on plants [15]. The experimental hardware’s impact on the proteomic responses accounted for 26.76% of the total variance, suggesting its crucial role in modulating the spaceflight-induced proteomic changes. This result resonates with previous studies such as that of Basu et al. [26] and Paul et al. [27], which suggested that spaceflight hardware could significantly influence plant physiology due to the variations in the microenvironments it creates. The influence of seedling age and light conditions on the proteomic responses (21.85% of the variability) is noteworthy, emphasizing the significance of these parameters in the context of space biology. This is especially evident with the distinct segregation of the BRIC PDFU experiment conducted on younger seedlings (3 days old) and in the absence of light, which is in line with known influences of light and developmental stage on plant physiology and gene expression [28,29].

Interestingly, the label techniques used for quantitative proteomics also emerged as a key factor influencing the data variance (20.15%). This underlines the potential effects that different physicochemical properties and labeling efficiencies of labels (iTRAQ and TMT) could have on peptide detection and quantification [30]. The unaccounted variance (16.58% in PC4 and 14.66% from the scree plot) indicates that other, yet unidentified, factors contribute to the observed proteomic variations. These factors could include, but are not limited to, genetic variation, subtle differences in plant handling, and latent differences in the growth environment. Unlike in the transcriptomics meta-analysis by Barker et al. [15], where hardware accounted for a lesser source of variation, the effects of hardware were amplified at the proteomics level. This resonates with the fact that mRNA expressions are usually transient with a pulse-like pattern in biological systems as a reactional response to environmental stimuli, unlike protein abundance, which usually establishes a new steady state in response to external stimuli, hence a stable adaptive response [10,31]. So far, limited attention has been placed on the molecular effects of hardware on plant spaceflight experiment outcomes, with choices of hardware usually dependent on funding agency specifications or research teams’ specific needs. This affects the reproducibility of spaceflight experimental data and reduces their overall confidence. Hence, we recommend that ground-based multiomics (transcriptomics, translatomics, and proteomics) research be conducted to assess the effects of existing spaceflight hardware on plants, leading to the establishment of operative guidelines on the choice of hardware for spaceflight experiments.

### 3.2. Protein Intersections and Functional Implications

Our analysis identified key proteins that are common across the four datasets, suggesting their potential role in Arabidopsis adaptation to spaceflight conditions. One such protein, the GLYCINE-RICH PROTEIN 1, which is involved in mRNA binding within the chloroplast [32], was found in all datasets, indicating its role might be pivotal in the response to spaceflight. Glycine-rich proteins are known to contribute to plant stress responses by regulating several RNA processes, such as alternative splicing, mRNA export, and RNA editing [33]. They have also been implicated in the acceleration of seed germination and seedling sprouting in low-temperature conditions [34]. Another common protein, the GLYCINE DECARBOXYLASE P-PROTEIN 1 (GLDP1), has a primary role in the mitochondrial glycine decarboxylase complex, which is crucial for photorespiratory metabolism in C3 plants [35]. The prominence of GLDP1 across all datasets could indicate an elevated photorespiratory metabolism in the spaceflight environment. This hypothesis aligns with previous findings that photorespiration is an essential component of the plant stress response and is tightly linked with photosynthesis, particularly under fluctuating light conditions [36]. Unlike the GLYCINE-RICH PROTEIN 1, which was upregulated in spaceflight in some of the datasets and downregulated in some others (Table 1)*,* the GLYCINE DECARBOXYLASE P-PROTEIN 1 (GLDP1) was downregulated across the four datasets (Table 1). The glycine cleavage system is particularly important for cellular energy production and detoxification processes. The GLDP1 enzyme complex converts glycine into serine while producing one molecule of CO_2_ and also transferring a methyl group to tetrahydrofolate (THF), forming 5,10-methylene-THF. This reaction is an essential part of the photorespiratory cycle, and it contributes significantly to one-carbon metabolism. Engel et al. [37] reported that the *gld1* Arabidopsis mutant had an incomplete lipoylation of H protein caused by defective mitochondrial lipoate biosynthesis. Lipoylation of the H protein is a crucial post-translational modification required for the function of several multi-enzyme complexes involved in energy metabolism, most notably the pyruvate dehydrogenase (PDH) complex and the glycine cleavage system. Consequences of the incomplete lipoylation of the H protein include disruption of cellular nitrogen balance due to the accumulation of glycine, impacting nucleotide synthesis, and cellular processes requiring methylation reactions. Downregulation of this protein across all four datasets may also indicate a reduced photorespiratory efficiency by the Arabidopsis plant in spaceflight. If energy is lost at a huge rate in spaceflight via photorespiration, the plants will require more energy input, which may result in the activation of pentose-phosphate shunt pathways as a faster source of NADPH to provide the cell with energy for reductive biosynthesis and detoxification of free radicals [38].

Several studies have noted a regulatory shift in Arabidopsis light-harvesting and photosynthetic genes [7,12,14], with Jie et al. [39] also reporting a significant decline in the capacity of plants to photosynthesize after exposing the seeds to the spaceflight environment. A major recommendation is to conduct an experiment to assess photosynthetic efficiency, yield, and shift in core regulatory proteins under different spaceflight conditions, starting with various simulated gravity and radiation dosages. This is essential due to the advent of new spaceflight hardware and as we prepare for exploration beyond the lower earth orbit. Future space missions should consider exploring the impact of spaceflight conditions on nucleotide production and one-carbon metabolic pathways in Arabidopsis mutants lacking functional GLDP1.

PATELLIN-1 (PATL1), a known regulator of auxin-mediated PIN1 relocation and plant development [17], was also shared across all datasets. Auxin signaling is a fundamental aspect of plant growth and development, and its role in gravity sensing and response is well documented [40,41]. The presence of PATL1 across all datasets suggests a possible alteration in auxin dynamics under spaceflight conditions, which is consistent with prior spaceflight studies that reported changes in auxin distribution and signaling [42,43,44]. The expression of auxin-related proteins in all four datasets is not novel; however, constructing the dynamics and modulators of auxin movement in plants in spaceflight has been a challenge. Future studies should consider the dynamics of auxin in PATL1 mutants under both simulated and real-spaceflight environmental conditions. PCAP1, also known as the MICROTUBULE DESTABILIZING PROTEIN, which is an oligogalacturonide-dependent phosphorylated protein, was also common to the datasets. PCAP1 is known for its role in the plant immune response through the activation of damage-associated molecular patterns (DAMPs) [18]. The fact that PCAP1 was shared across all datasets could suggest an altered immune response in Arabidopsis under spaceflight conditions. Several molecular studies have reported enrichment terms involved in pathogenesis, response to disease, fungi, and bacteria in plant spaceflight datasets [15,45]. The filament-severing ability of PCAP1, which is anchored to the plasma membrane, might also be indicative of disrupted intracellular transport via the vesicular transport route. The protein–protein interaction network analysis (Figure 3A) highlighted potential interactions among these proteins. Notably, the interactions between these proteins might underlie a broader coordinated response to the spaceflight environment, involving shared pathways such as protein modifications, microtubule polymerization regulation, and unidimensional growth.

The involvement of central carbon metabolism and amino acid biosynthesis pathways across the four datasets is expected given their fundamental roles in plant growth and development (Figure 3C). Under spaceflight conditions, these pathways might experience alterations due to changes in energy demand, stress response, and growth patterns. The glyoxylate cycle, which bypasses the decarboxylation steps of the citric acid cycle to convert lipids into carbohydrates, is known to be crucial under stress conditions when carbohydrate availability is limited [46]. The involvement of this pathway across all datasets could indicate a metabolic shift in Arabidopsis under spaceflight conditions, possibly due to altered resource availability or energy requirements. Gluconeogenesis, the process of generating glucose from non-carbohydrate precursors, is another stress-related pathway. Its role could be associated with maintaining glucose homeostasis in the spaceflight environment, which might pose significant energetic challenges. A limited effort has been dedicated to studying the energetics of plants in response to the spaceflight environment. This might be due to the inherent inaccessibility of space stations to scientists. We recommend that photosynthetic studies of plants under simulated spaceflight conditions be coupled with studies on the dynamics of energetics using mutants deficient in genes in energetic metabolic pathways.

### 3.3. Integration of Light Signals in Spaceflight Leads to Altered Metabolism

The 109 proteins identified at the intersection of the BRIC LED and BRIC PDFU datasets (Figure 5A) are indicative of the baseline response to spaceflight conditions, irrespective of light availability. The functional enrichment of shared proteins to pathways associated with ribosomal activities, the S-adenosylmethionine (SAM) biosynthetic process, carbon fixation, glyoxylate, and dicarboxylate metabolism, and response to toxic substances could provide key insights into the adaptation strategies employed by Arabidopsis under the unique stress conditions presented by spaceflight. SAM is a universal methyl group donor involved in a myriad of methylation reactions that affect nucleic acids, proteins, lipids, and secondary metabolites [47]. Methylation processes play crucial roles in plant stress responses, affecting gene expression, protein function, and signal transduction. The enrichment of this pathway suggests that changes in methylation patterns might be part of the Arabidopsis response to spaceflight stress. This is consistent with the epigenomic analysis of Paul et al. [48] and Mingqi et al. [49], which identified DNA methylation and the roles of methyl transferases as important plant adaptation mechanisms in spaceflight. The enriched pathways of the intersection proteins, to which we alluded solely to the influence of the spaceflight environment, highlight the multifaceted strategies that Arabidopsis might employ to cope with the unique spaceflight environment, involving not just specific stress-response mechanisms but also fundamental metabolic and cellular processes. We recommend that wet laboratory experiments be conducted on proteins observed in this intersection to confirm their roles and dynamics in Arabidopsis spaceflight response.

The incorporation of light in the BRIC LED experiment elicited a distinct proteomic signature, enriched in photosynthesis-related pathways (Figure 6A), underscoring the indispensable role of light in driving photosynthesis, a pivotal process for energy provision and carbon fixation in plants. On the other hand, proteins unique to the BRIC PDFU dataset were notably enriched in amino sugar and nucleotide sugar metabolism pathways (Figure 6B). These pathways are key contributors to the biosynthesis of structural polysaccharides, glycoproteins, and glycolipids, essential components of the plant cell wall and membrane [50]. The enrichment of these pathways might imply an adaptive restructuring of the cell wall and membrane in the absence of light. Etiolated seedlings have been reported to have thinner cell walls with low calcium and pectin content [51].

### 3.4. Disentangling Microgravity from Other Plant Spaceflight Responses

By contrasting the EMCS 1 *g*/1 *g* dataset, where microgravity effects are nullified, with the proteomes of the intersection of BRIC LED and BRIC PDFU experiments, we isolated proteins potentially related to the response to microgravity and those due to other factors in the spaceflight environment. The intersection of this comparison is believed to be proteins expressed in response to spaceflight factors other than microgravity. SUC1, one of the six proteins at this intersection, is integral to carbohydrate translocation, a process that is central to the plant’s energy management and metabolic regulation and could be crucial in handling the energy-demanding stress conditions in spaceflight. The presence of CYSTEINE SYNTHASE 1, a regulator of redox homeostasis and the synthesis of glutathione, also suggests that not only microgravity is responsible for the oxidative damage experienced by plants in space. The glycolysis and pentose phosphate pathways enriched solely due to microgravity (Figure 7C) are central to energy production and the generation of reduced power in the form of NADPH, respectively. An increased emphasis on these pathways might imply a heightened demand for energy and reducing equivalents under microgravity conditions. Similarly, the enrichment of proteins involved in glutathione, ascorbate, and aldarate metabolism suggests an elevated role for antioxidant mechanisms in microgravity. These had been confirmed by simulated microgravity studies [52,53].

Our exploration of proteomics datasets from Arabidopsis spaceflight experiments provides novel insights into the complexity of plant proteomic responses to the spaceflight environment. The multifaceted adaptations implicated involve fundamental cellular processes such as protein synthesis, methylation, carbon fixation, and detoxification. This shows the extent of molecular rearrangements that Arabidopsis plants undergo when faced with the unprecedented challenges of spaceflight, such as microgravity and cosmic radiation. While our study provides a significant step forward in understanding plant responses to spaceflight, it also underscores the need for further research. Each of the revealed adaptations, whether it is the enhanced ribosomal activities, alterations in carbon metabolism, or augmentation of stress-response mechanisms, warrants wet-lab investigations. The combination of such experimental data with advanced computational modeling could help elucidate the full scope of plant responses to spaceflight and inform strategies for successful space farming.

## 4. Materials and Methods

### Accessing and Analyzing Individual Proteomics Datasets

To ensure the greatest degree of comparability between the datasets, raw LC/MS spectra peak representing the spaceflight and ground control proteomics datasets (.RAW format) for BRIC PDFU and BRIC LED spaceflight experiments were accessed from the NASA GeneLab data depository with accession numbers GLDS 38 (https://osdr.nasa.gov/bio/repo/data/studies/OSD-38, accessed on 9 April 2023) and OSD 522 (https://osdr.nasa.gov/bio/repo/data/studies/OSD-522/preview/w5Q5ElZE-Wy6DDS1ZFzeRjBAPuKss-x0, accessed on 16 July 2023), while the ABRS dataset was accessed from the PRIDE Archive proteomics data repository with accession number PXD001179 (https://www.ebi.ac.uk/pride/archive/projects/PXD001179, accessed on 16 July 2023). The EMCS experiment datasets (0 *g*/1 *g* and 1 *g*/1 *g*) were not deposited in any accessible data repository, but the ready-analyzed peptide and protein count data was attached as a Supplementary Materials to the primary publication.. The description of the experimental setup for each of the datasets obtained is accessible from their individual repository. The raw LC/MS spectra peak from BRIC LED, BRIC PDFU, and ABRS experiments were fed into MaxQuant v2.4.2.0 (downloaded April 2023) [54], a quantitative proteomics software package for analyzing large mass spectrometric data sets. MaxQuant supports label-free quantification and all main labeling techniques, including SILAC, Di-methyl, TMT, and iTRAQ. The peak intensity (counts) for each spaceflight and ground control replicates per dataset were analyzed using Perseus v4.0 (downloaded April 2023) [55] for differential expression and estimation of the *p*-value and the Benjamini-Hochberg [56] adjusted *p*-value to control for false discovery rate. In our proteomics analysis pipeline, we generated adjusted *p*-values, commonly referred to as q-values, that account for the false discovery rate (FDR) associated with multiple testing. It’s worth noting that our FDR is maintained at <0.01 to ensure high statistical stringency. However, to maintain an inclusive analysis, we have chosen not to apply high fold-change cutoffs. This approach ensures that we capture the broadest range of differentially expressed proteins, allowing for a more comprehensive understanding of proteomic alterations. The adjusted *p*-value and our FDR of 0.01 were quite strict, and we are careful not to lose biologically significant proteins. Some proteomics studies [57,58] also advised a lower fold change threshold when comparing labeled and non-labeled datasets. The four datasets consist of both labeled and unlabeled samples. The labels used were also different across the labeled samples. Hence, we prioritized FDR and adjusted the *p*-value over the log fold change.

Perseus v4.0 Differential Expression (DE) analysis plugins for EdgeR and Limma R/Bioconductor packages were installed for Rstudio v4.2.3. R codes for the downstream analysis are available as Supplementary File S3. The log2 fold change was calculated for the EMCS dataset counts (Supplementary File S1). Protein annotations for each dataset were assigned using the org.At.tair.db (v3.8.2), STRINGdb (v1.24.0), and PANTHER.db (v1.0.4) packages from the Perseus v4.0 platform. A threshold of adj *p*-value ≤ 0.05 was established for significantly expressed proteins, with a false discovery rate (FDR) ≤ 0.01.

Online analytical tools such as ShinyGO v0.76 [20], Metascape v3.5.20230501 [21], and InteractiVenn [25] employed in the data analysis are noted in the text and figure legends. The analysis of the dissimilarity index, multidimensional scaling analysis (MDS), principal component analysis (PCA), and hierarchical clustering were performed with the R programming language (https://www.r-project.org/about.html, accessed on 1 April 2023) within the commercially available Rstudio v4.2.3 IDE. The Rstudio analytic packages and codes used are available in Supplementary Materials.

## Figures and Tables

**Table 1:** Proteins identified in all four Arabidopsis spaceflight proteomics datasets.

Gene ID	Symbol	Name	ABRS	EMCS	BRIC PDFU	BRIC LED
Proteins shared by all the datasets			Log 2 Fold Change (L_2_FC)			
AT1G27090		Glycine-rich protein	−0.66	0.29	0.23	−0.37
AT1G72150	PATL1	Patellin-1	0.71	−1.318	−0.17	0.57
AT4G20260	PCAP1	Plasma-membrane-associated cation-binding protein 1	0.73	−0.79	−0.44	0.49
AT4G33010	GLDP1	Glycine decarboxylase P-protein 1	−0.96	−1.27	−0.35	−0.47
AT5G26260		Leucine-rich repeat receptor-like kinase1	0.59	0.56	−0.414	0.8
Proteins shared by EMCS, BRIC PDFU, and BRIC LED but not ABRS						
AT1G74470	CHLP	Geranylgeranyl diphosphate reductase		0.37	0.54	−0.54
AT3G16470	JAL35	Jacalin-related lectin 35		−1.53	−0.46	−0.35
AT3G22640	PAP85	Vicilin-like seed storage protein		−0.901	0.02	3.31
AT5G19550	ASP2	Aspartate aminotransferase		1.13	−0.14	0.31
AT5G37600	GLN1-1	Glutamine synthetase cytosolic isozyme 1-1		0.82	−0.06	0.23

## Data Availability

Data sets analyzed in this paper are from the NASA GeneLab data depository with accession numbers GLDS 38 (https://osdr.nasa.gov/bio/repo/data/studies/OSD-38, accessed on 9 April 2023), OSD 522 (https://osdr.nasa.gov/bio/repo/data/studies/OSD-522/preview/w5Q5ElZE-Wy6DDS1ZFzeRjBAPuKss-x0, accessed on 16 July 2023), and the PRIDE Archive proteomics data repository with accession number PXD001179 (https://www.ebi.ac.uk/pride/archive/projects/PXD001179).

## Supplementary Materials

The following supporting information can be downloaded at https://www.mdpi.com/article/10.3390/ijms241914425/s1.
